# Understanding Perceptions of Hepatitis C and Its Management Among People with Experience of Incarceration in Quebec, Canada: A Qualitative Study Guided by the Common Sense Self-Regulation Model

**DOI:** 10.3390/v16121910

**Published:** 2024-12-12

**Authors:** Andrea Mambro, Sameh Mortazhejri, David Ortiz-Paredes, Andrea Patey, Guillaume Fontaine, Camille Dussault, Joseph Cox, Jeremy M. Grimshaw, Justin Presseau, Nadine Kronfli

**Affiliations:** 1 Centre for Outcomes Research and Evaluation, Research Institute of the McGill University Health Centre, Montreal, Canada; andrea.mambro@muhc.mcgill.ca (A.M.); david.ortiz.paredes@umontreal.ca (D.O.-P.); camille.dussault@muhc.mcgill.ca (C.D.); joseph.cox@mcgill.ca (J.C.); 2 Centre for Implementation Research, Methodological and Implementation Research, Ottawa Hospital Research Institute, Ottawa, Canada; smortazhejri@ohri.ca (S.M.); patey@ohri.ca (A.P.); guil.fontaine@mcgill.ca (G.F.); jgrimshaw@ohri.ca (J.M.G.); jpresseau@ohri.ca (J.P.); 3 School of Epidemiology and Public Health, University of Ottawa, Ottawa, Canada; 4 School of Rehabilitation Therapy, Queen’s University, Kingston, Canada; 5 Centre for Clinical Epidemiology, Lady Davis Institute for Medical Research, Jewish General Hospital, Montreal, Canada; 6 Ingram School of Nursing, Faculty of Medicine and Health Sciences, McGill University, Montreal, Canada; 7 Kirby Institute, University of New South Wales, Sydney, Australia; 8 Department of Medicine, Division of Infectious Diseases and Chronic Viral Illness Service, McGill University Health Centre, Montreal, Canada; 9 Department of Epidemiology, Biostatistics and Occupational Health, School of Population and Global Health, Faculty of Medicine and Health Sciences, McGill University, Montreal, Canada; 10 Department of Medicine, University of Ottawa, Ottawa, Canada; 11 School of Psychology, University of Ottawa, Ottawa, Canada

**Keywords:** people with experience of incarceration, hepatitis C virus, CS-SRM, qualitative research, implementation science

## Abstract

Hepatitis C virus (HCV) disproportionately affects certain sub-populations, including people with experience of incarceration (PWEI). Little is known about how perceptions of HCV and treatment have changed despite simplifications in testing and treatment in carceral settings. Nineteen semi-structured interviews were conducted with people living with or having a history of HCV infection released from Quebec provincial prison. Interviews were guided by the Common Sense Self-Regulation Model (CS-SRM) and aimed to explore cognitive and emotional representations of HCV and coping strategies. Among the 19 participants, seven (37%) were diagnosed with HCV in prison and 14 (74%) had previously received HCV treatment. Participants’ HCV illness perceptions were influenced by fear (of HCV transmission, death, and the well-being of family) and stigma (related to HCV, injection drug use, and incarceration). While some sought education and social and professional support, others self-isolated or engaged in high-risk behaviors to cope. Despite advances in HCV treatment, PWEI continue to experience various forms of stigma and fear surrounding their HCV diagnosis, resulting in delayed HCV care. These findings provide insights into how prison-based healthcare providers can better utilize HCV illness perceptions to evaluate willingness to engage in HCV care among PWEI.

## 1. Introduction

Chronic hepatitis C virus (HCV) infection disproportionately affects certain sub-populations, underscoring the importance of targeted efforts among priority groups if the World Health Organization (WHO) elimination efforts are to be achieved by 2030 [1,2]. Canada has defined people with experience of incarceration (PWEI) as a priority population for national HCV elimination efforts [3]. People who are incarcerated in Canadian prisons represent 10% of the overall HCV burden in Canada [4]. Most recent statistics by the Public Health Agency of Canada show that 1 in 10 people in prison—or 38,000 individuals—have been exposed to HCV, pointing to the urgent need to improve uptake of viral hepatitis services and care in prison [4]. Given the interface between carceral settings and surrounding communities, as well as high turnover and recidivism rates among incarcerated individuals, optimizing HCV care in all prisons globally is paramount for HCV elimination [5,6,7,8,9,10,11,12,13].

An extensive body of work exists on ways in which people who inject drugs with chronic HCV conceptualize HCV [14,15,16,17,18,19]. This research suggests that an individual’s understanding of their illness affects their willingness to get screened for HCV, to consider initiating treatment, and to complete treatment once started. Much less research has been published on ways in which PWEI with chronic HCV perceive their infection and its management in prison or following release. While most research has been published in the interferon era [20,21,22], much less is known in the direct-acting antiviral era despite simplifications in testing and treatment [23,24,25]. A better and current understanding of how PWEI perceive their chronic HCV infection may offer insights into how healthcare providers in prison, and in surrounding communities, can better understand and utilize HCV illness perceptions to evaluate willingness to engage in HCV care and treatment among PWEI [18].

**Figure 1:**
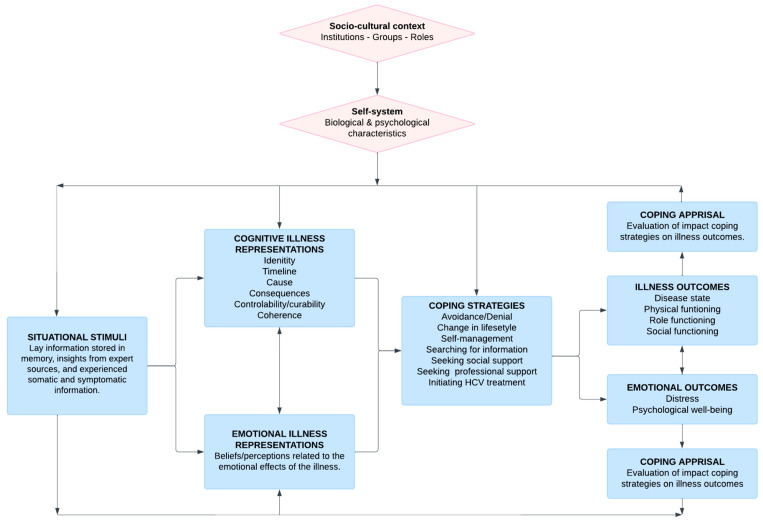
The Common Sense Self-Regulation Model (CS-SRM), adapted from Hagger and Orbell (2022).

The Common Sense Self-Regulation Model (CS-SRM)—or illness perception model—is a well-recognized social cognition approach [26,27,28] that seeks to understand how individuals’ perceptions of “illness threats” guide their behaviors (coping strategies) in addressing these threats. Figure 1 presents the CS-SRM and its main components [26]. According to this model, people develop beliefs and emotions about an illness, known as illness representations, based on information stored in memory and acquired through external (e.g., healthcare professionals, media) or endogenous (e.g., symptoms experienced) sources. Illness representations are categorized into two major groups: cognitive illness representations and emotional illness representations. Cognitive illness representations represent individuals’ logical beliefs about an illness and can be further subdivided into six categories: identity, timeline, cause, consequences, controllability/curability, and coherence. Emotional illness representations represent individuals’ reflections on their emotional responses to the illness. Based on these illness representations, individuals develop, enact, appraise, and update a set of coping strategies. New experiences may lead to new illness representations, which in turn generate new coping strategies or update existing ones [29,30].

The CS-SRM is a dynamic framework which can offer insights into the evolving P perceptions of HCV and its management among PWEI. To our knowledge, the CS-SRM has never been applied to PWEI. That being said, previous studies among PWEI have demonstrated several barriers to HCV treatment initiation, including but not limited to ongoing injection drug use, limited access to harm reduction services, and HCV-related stigma [31,32,33,34,35,36,37]. Conversely, a strong motivator for the completion of HCV treatment in prison was the reduced number of competing priorities—many of which take precedence post-release [38,39,40]. These studies underscore that PWEIs’ illness perceptions of HCV play a role, among many other factors, in treatment initiation in prison. Similar studies are needed in Canada given the disparate political, structural, and organizational factors that exist among carceral settings worldwide, limiting the generalizability of previous studies [41]. We thus aimed to describe perceptions of HCV and its management among PWEI in Quebec, Canada.

## 2. Materials and Methods

### 2.1. Study Design

We conducted a theory-informed qualitative study using semi-structured interviews based on the CS-SRM to understand how PWEI in Quebec provincial prisons perceive HCV and how their beliefs and perceptions shape their management of their illness.

### 2.2. Setting and Participants

There are 16 provincial prisons in Quebec, and they housed approximately 4412 sentenced individuals in 2022/2023 [42]. The mean duration of incarceration in Canadian provincial prison is 28 days [43]. Although we initially intended to recruit individuals in Quebec provincial prisons, all non-essential prison-based research was halted from March 2020–2022 due to the COVID-19 pandemic [44,45]. Consequently, we opted to recruit individuals recently released from prison rather than those currently sentenced in prison.

We recruited individuals aged ≥ 18 currently or previously living with chronic HCV who were incarcerated in one of Quebec’s 16 provincial prisons within the previous 12 months and who were able to consent to study participation in either English or French. Potential participants were recruited using a convenience sampling strategy from CACTUS Montréal, a large urban harm reduction community organization dedicated to the prevention of sexually transmitted and blood-borne infections in Montréal. In 2020–21, 70,939 people visited CACTUS Montréal to use its various programs (e.g., needle and syringe programs, supervised injection sites, and the social involvement program for people who use drugs) [46]. Data saturation in qualitative research is an important indicator of quality and trustworthiness. In this study, we gauged saturation by whether new themes emerged after each interview. To ensure an adequate sample for content validity, we applied the 10 + 3 decision rule [47], whereby we aimed to conduct 10 initial interviews, followed by an additional three interviews until no new themes emerged.

### 2.3. Interview Guide

The interview guide was developed by the study team using CS-SRM to describe perceptions of HCV and its management among PWEI in Quebec provincial prisons. The different dimensions of the CS-SRM are presented in Table 1, with their definitions and examples from the interview guide. The interview guide included one to three questions for each of the CS-SRM dimensions, and for the latter, we explored whether individuals’ perceptions underwent changes over time. The interview guide was not pilot-tested.

### 2.4. Data Collection

Potential participants were identified and approached at CACTUS Montréal by a member of the research team who outlined the objectives of the study. Those who expressed interest in participating were given the information and consent form (in English or French) in person. Participants provided written informed consent prior to participation.

All interviews were conducted in person by two members of the research team (AM and DOP) in English or French. All interviews were audio-recorded, and recordings were transcribed verbatim, translated to English, and anonymized simultaneously. Transcripts were not revised or verified by participants, but they were verified for accuracy by AM. All interviews were included in the analysis.

Participants received gift cards of CAD 50 for their study participation [48]. This study was approved by the McGill University Health Centre Research Ethics Board (#2021-7453).

### 2.5. Data Analysis

All transcripts were independently double-coded, using NVivo 11 software, by two members of the research team (AM and SM). Coders met after coding the first four transcripts to discuss and resolve any discrepancies and to develop a uniform coding scheme. A deductive approach was used for coding, followed by a thematic analysis using CS-SRM dimensions. Discrepancies were resolved via consensus.

## 3. Results

From 17 January to 14 December 2022, 19 participants (11 (58%), 5 (26%), and 3 (16%) who self-identified as cis male, cis female, and transwomen, respectively) were interviewed. All interviews were conducted in French and lasted an average of 58 min (range: 34–88 min). Interviews consisted of two separate research questions, one of which is the focus of a separate manuscript. The median age of participants was 48 years old (range: 33–59 years). The highest level of education among most participants was high school (n = 15); four individuals had completed a university degree. Of the 19 participants, 7 (37%) were diagnosed with HCV in prison. Fourteen had received treatment for HCV, among whom, six were cured and three had received treatment in prison; one individual had been spontaneously cured. Four participants had not yet received direct-acting antivirals: one was awaiting treatment initiation, while the other three were not yet ready to start treatment.

### 3.1. Cognitive Illness Representations Regarding HCV Infection

#### 3.1.1. Identity: Label and Symptoms Secondary to HCV Infection

Participants’ perceptions of their identity in relation to chronic HCV infection depended on their awareness of symptoms. Most participants had not noticed or experienced any HCV-related symptoms until diagnosed, underscoring a disconnect between the presence of chronic HCV infection and their perception of illness.

*“Well, I had done tests at some point and the doctor told me that I had hepatitis C, but I didn’t have symptoms before that.”*

*(P11)*

*“I don’t know, it seems to me that the symptoms, I didn’t have that many. It’s because when you get stoned, you don’t know”*

*(P17)*

Despite this, some participants described a range of symptoms that they associated with their HCV infection, including fatigue, gastrointestinal (nausea, vomiting, loss of appetite, and changes to bowel movements), fever or flu-like-symptoms, yellow eyes, and “liver” pain. This reflects participants’ attempts to interpret and attribute their physical experiences to their illness. For some, this process led to the formation of an illness identity characterized by uncertainty and concern. A few participants reported non-specific symptoms, including headaches, dizziness, and weight loss, which they often struggled to attribute to HCV.

*“That’s it, a lot of fatigue. I was always asking myself what I had, like my body was sore, like a big, big flu, but like I would have been beaten up, you know? Like … messed up, but from the inside. Like bruises, but from the inside. The body was all «aouh, aouh, aouh», you know? I don’t know why. That’s what the symptoms were, you know? A hard time going forward.”*

*(P15)*

*“I was dizzy, vomiting, I was always tired, I had fever too. That’s about it. And my liver was constantly hurting.”*

*(P6)*

#### 3.1.2. Timeline: Beliefs About the Onset, Duration, and Fluctuations of HCV Infection

It was difficult to clearly understand the timeline of the disease, as many participants had no symptoms. For those who had experienced symptoms, these lasted anywhere between one week and five years before they sought screening and care. The symptoms fluctuated in severity throughout the day for most participants. Some of the participants without symptoms believed their disease was due to an incident (e.g., needlestick injury or fight) that occurred 10 to 20 years before the diagnosis was made.

#### 3.1.3. Cause: Beliefs About the Predisposing Risk Factors of HCV Infections

All participants believed they knew how they had acquired HCV, attributing the infection to specific high-risk behaviors. The most common cause was perceived to be the sharing (at times, accidental) of unsterile needles during injection drug use or unsterile tattoo equipment—activities that occurred either in prison or in the community. However, a few participants also mentioned other potential sources, such as sexual intercourse, street fighting, cutting rituals, and unsanitary prison conditions. The most frequently reported cause was the sharing of unsterile needles, often in situations where access to clean needles was limited.

*“The first time, it was in 2000, and it wasn’t a needle exchange, it was a needle error, because I placed my syringe beside the one for the other guy that I was injecting myself with, a friend, and he said, he said “You made a mistake”, he said, “if you took mine”, he said, “I have hepatitis C”. And, it was true, and I had it. An accident.”*

*(P13)*

*“Before I used to inject myself. And we exchanged syringes in prison, yeah, because there aren’t many! So that’s it.”*

*(P6)*

Another important cause identified by participants was the use of unsterile tattoo equipment, often during incarceration.

*“It was during my second incarceration in 1996. I got tattooed, and the person that tattooed me had hepatitis C and he didn’t clean his stuff and at that time I was young, and I didn’t think about that, those diseases.”*

*(P4)*

Some individuals perceived cigarette smoking and unsanitary spaces in prison as causes of HCV transmission.

*“Listen, when you smoke a cigarette with someone, you can catch hepatitis C. It’s no joke!”*

*(P2)*

*“It’s like if, because of the prison, because the prison is really dirty, it’s like the rooms are disgusting. It’s like they never clean. They never clean the room, never sweep. It’s like the sink is always disgusting. When you drink the water, the water doesn’t taste good. It’s really… the right expression, my friend, I would not even put a dog in there. It’s like it’s disgusting.”*

*(P1)*

Lastly, one individual explained that drinking alcohol, in combination with other substances, can make one more susceptible to high-risk behaviors.

*“If I combine alcohol with using, I don’t think anymore. [Hu-hum] I take risks that I would not take if I was sober.”*

*(P12)*

#### 3.1.4. Consequences: Beliefs About the Impact(s) of HCV Infection

The perceived consequences of living with HCV varied among participants. While approximately one-third of participants believed that HCV had no major consequences for their lives, many reported significant effects on their interpersonal relationships, mental health, and daily functioning. The fear of transmitting the virus, combined with the stigma associated with HCV, often led to social isolation and altered self-perception. Additionally, concerns about the future, especially regarding life expectancy and the ability to care for loved ones, were prominent.

The effects of HCV on inter-personal relationships were voiced by many. Participants explained that they had limited intimate relationships and socializing due to fear of transmitting the virus to others but also to avoid prejudice and stigma that often accompanies such fears.

*“It’s been […] 2 years for sure that I haven’t had any sexual relations with anyone. I don’t know, I’m sort of blocked. I am completely blocked. […] I didn’t really accept to be in public while believing that I could contaminate someone. But really, one of the big consequences, it’s really the isolation. It was long before I could get out of it. Really a long time before I got out of it.”*

*(P12)*

*“People rejected me, they weren’t the same with me, they didn’t talk to me the same as before, they were scared to catch it.”*

*(P6)*

*“I’m afraid that they leave me, it’s fear especially, of being abandoned, being put aside because of that disease.”*

*(P5)*

*“Because to start with, there is so much prejudice on that, hepatitis C, right away you are a junkie, you are not clean, someone that doesn’t take care of themselves, a dishonest person. You are a criminal, basically, if you have hepatitis. It’s everything we deserve, according to the « normal citizens »—like we say in brackets—it’s prison, if we have hepatitis. That’s how normal citizens see us.”*

*(P3)*

*“I was pushed aside in my village, they saw me as a walking disease, they were scared of me, they wouldn’t let me into certain places. My family didn’t want to take a drink out of a glass after me, and I couldn’t touch anything. My mother threw away dishes because my stepfather said “Ark! Your son touched that!” They had to throw it away because they didn’t want to wash it.”*

*(P8)*

Some expressed concerns about their future, including a fear of dying due to the impact of HCV on their life expectancy. Parents with HCV worried about transmitting the virus to their children and were concerned about who would care for them when they could not. Parents also struggled to care for their children while managing their illness, highlighting the dual burden of disease and caregiving responsibilities.

*“It really was the worst news of my life. Because like I told you, I am athletic, I am someone that takes care of himself physically speaking, I’m in good shape…and I took it hard, because you know, me, I want to live to be old, I want… I spent most of my life in prison, I have time to catch up on when I’m outside…”*

*(P11)*

*“Because I had children that were adopted and I hadn’t found them yet, I didn’t know how I would announce to them that I was going to die.”*

*(P3)*

*“My family, plus my son, you know. I don’t want him to… I don’t want him to touch me when I have blood, let’s say, I don’t want him to touch me…I was afraid. I didn’t want him to catch it.”*

*(P18)*

*I: “You said your most important concern was your baby, your child?”*

*P: “Yeah. I had a son and I had nobody to take care of him (cries), so I fought every day, if I was sick or not.”*

*(P16)*

The lack of energy and sleep problems due to HCV affected daily activities and work life:

*“Because there were tasks that I didn’t function as well and that I would postpone them. I didn’t do everything that I wanted to do, that I had to do, yeah, that was pretty much the disadvantage.”*

*(P13)*

Forgetfulness, difficulty processing information, and loss of self-confidence and purpose in life were predominant concerns. These cognitive and emotional challenges were particularly palpable at the time of diagnosis, when the reality of the diagnosis was made apparent:

*“And, at some point, when they announced it to me, like it or not, I was forgetting things, you know? We call that the “shock of finding out.” Well, you know, we forget things afterwards, you know? [Yeah] So, there’s information that I kept, but there is other information that didn’t get in, because I was too busy to even think about my future, and this, and that.”*

*(P11)*

*“It affects your self-esteem, it affects the way you see life, you know, at the time? Right now, I don’t care! When I accepted the disease and everything, ok.”*

*(P3)*

#### 3.1.5. Controllability/Curability: Beliefs Regarding the Controllability and Curability of HCV Infection

Almost all participants knew that HCV could be cured with treatment, while others explained that there was a possibility that HCV could be healed by itself. These beliefs translated into a perception of HCV as a controllable condition, which contributed to a sense of optimism and reduced anxiety about living with and managing the disease.

*“I’m not too alarmed because there are treatments, I could still heal pretty well. It’s rare, apparently, today that people die from it.”*

*(P10)*

*“Today, for me, I say to myself “There’s nothing to it! If it comes back, I have the treatments and it will be easier than back then because back then we took a lot of medication.”*

*(P5)*

*“I know that it can get worse if I’m not treated, but there’s a 25% chance that it will heal on its own, apparently.”*

*(P13)*

#### 3.1.6. Coherence: Understanding HCV Infection

Participants’ understanding of HCV infection varied, with many initially possessing limited knowledge about the disease. Over time, and through interactions with healthcare providers, their understanding of HCV improved. For some, knowledge gaps persisted, which influenced how they perceived and managed their condition.

Many participants initially had limited understanding of HCV, which led to fear and anxiety. This lack of knowledge often resulted in exaggerated perceptions of disease severity:

*“I didn’t know hepatitis C well, it was like they were telling me that “I was going to die” …, because since I didn’t know about it, I had the impression that they were trying to announce to me that I was going to die soon, because I didn’t know it well.”*

*(P12)*

*“I didn’t know, I wasn’t aware of any of it, the fibrosis that… that they found. I didn’t know. They showed me the liver that deteriorated, and with… livers that are finished, livers, really damaged.”*

*(P13)*

Over time, participants’ understanding of HCV improved, particularly after conversations with healthcare providers or through personal experiences. This shift in knowledge is likely explained by changes in treatment options and access, resulting in shifts in disease perception.

*“I was completely ignorant, naïve, not conscious, I never thought that I would catch that in my lifetime. I didn’t even know that it could be caught that way. I was really naïve because I did a lot of prison, eh? So, I didn’t understand, so now, I understand!”*

*(P6)*

*“Ah, now, today, the doctor, with all the information we have, the doctor would tell me that I have hepatitis C and I would start to laugh. Right now, you know, hepatitis C problems, it’s the same as a flu, you know? It can be treated. That’s how I see it now. But 10 years ago, it wasn’t the same thing.”*

*(P3)*

There was some confusion among participants about HCV-related symptoms, as those who had experienced symptoms had initially attributed them to other diseases or conditions (e.g., food poisoning, bacterial infections, and medication side effects). Over time, they realized that their symptoms were due to HCV. This misattribution reflects the challenges in recognizing and diagnosing HCV based on its symptoms, which are often nonspecific and can be mistaken for other health conditions.

*“I always thought that it was either the food, something like that, but I found out that no, it wasn’t… it was hepatitis.”*

*(P10)*

*“For me, it was like I thought that I caught a bad bacteria.”*

*(P1)*

*“I’m always exhausted, so I don’t know if it’s that [Hepatitis C] or if it will be other things. I don’t know what it can be. Will it be an endocarditis? Will it be the anemia? Will it be the hepatitis?”*

*(P17)*

*“It’s been two and a half years that I’ve been taking PrEP, about. And in the beginning, because PrEP started roughly a bit after that, I thought that PrEP contributed to it. In the beginning, I thought that PrEP made it that it wasn’t stopping, that it was contributing to the diarrhea, but it wasn’t that because it got all sorted out at one point.”*

*(P12)*

There were misconceptions about HCV transmission and prevention methods. Two participants believed that HCV could not be transmitted during pregnancy or sexual intercourse. When we asked how HCV can be prevented, one participant replied:

*“Take vaccines for hepatitis C also, shots for hepatitis C, antibiotics, be mindful to it and ask your doctor next time for the hepatitis C vaccine.”*

*(P1)*

While most participants were aware that treatment is available for HCV, a few were unsure about cure rates and the risk of re-infection.

*“With everything that I read, all that, it makes it that it takes so much time to cure that I more or less believe it. So, it’s now that… yes, it works, but at which… does it work for everyone? Does it get cured quickly, does it get cured slowly? …Does it get cured and I can reinfect myself? Because, obviously, I did the same stupid thing again a few months ago where I injected myself with someone else’s syringe! So, can I put myself at risk after? When you use, you don’t realize everything.”*

*(P12)*

Interestingly, some participants compared HCV to HIV to make sense of the disease.

*“… for me, hepatitis C and HIV, it’s the same thing, that is to say, “it’s two things that can’t be cured”. So… it was like a disaster.”*

*(P12)*

*“When I found out that my body was healing it, I was happy, very happy, because for me,*

*hepatitis C is like AIDS, you know.”*

*(P16)*

*“It’s more serious hepatitis C because it’s more harmful than HIV… apparently.”*

*(P18)*

### 3.2. Emotional Illness Representations Regarding HCV Infection

A wide range of emotions were experienced during the course of the illness. The initial diagnosis often triggered emotions such as fear, sadness, and shock. As participants processed their diagnosis, they experienced anger, shame, guilt, and even relief once they learned that the illness could be treated. These emotional representations played a significant role in how participants coped with their condition and perceived their own health.

The first emotional response to a diagnosis of HCV for many participants was fear, particularly fear of dying, in addition to sadness and shock:

*“It’s the fear that kicked in, I was really scared of dying”*

*(P11)*

*“I would say that mentally, I was sort of depressed. I was having a depression, too, I believe. I had dark thoughts, I even thought of suicide in those moments.”*

*(P11)*

*“I don’t know how to say this, but we were there and “Ah, shit!”, you know? Not me, it can’t be true?! Fuck! How could I have caught that? And how do we catch this, first of all?”*

*(P5)*

Beyond the initial shock, participants dealt with emotions such as anger, shame, guilt, and hatred. Some shared that they felt naïve, worthless, dirty, and diminished. These feelings were deeply entangled with their sense of self-worth and identity:

*“So, shame and guilt, it’s the two first things. I’m ashamed of that, I would not tell that to anyone. I don’t talk about that to everyone.”*

*(P12)*

*“I didn’t feel capable, I didn’t feel worthy of people, you know? I really didn’t feel worthy.”*

*(P15)*

*“First of all, there was my self-confidence. It really declined. We feel… completely diminished as a person, we feel… less… even when they announce it to us, somewhere, it’s psychological…”*

*(P11)*

A few described feeling betrayed by those who may have transmitted the infection to them, adding to their emotional distress.

*“I also felt betrayed concerning the tattoo and I wasn’t proud of myself for not having been more careful.”*

*(P4)*

*P: “I find it important to take care of my health, and it really bothered me to find that [diagnosis] out. And I felt betrayed by that woman.”*

*I: “What woman is that?”*

*P: “I picked her up on the street, like that.”*

*(P11)*

Some individuals expressed concerns about the possibility of transmitting the virus to others, which often led to social withdrawal:

*“I left my job not long after that [diagnosis], I felt like trash, and I was afraid because I was working in customer service, and I was worried… I’m the one who decided to leave, yes, but… I didn’t really accept to be in the public while believing that I could contaminate someone.”*

*(P12)*

Despite all of these negative emotions, most participants were relieved once they learned that their illness could be treated.

*“I jumped for joy when he told me that [treatment was available]! I jumped for joy and then I took a big puff of crack! … I was really happy”*

*(P3)*

*“It [treatment] gave me hope, a smile. Just the fact of saying “At least, I can be cured, I’m not stuck with that for the rest of my life”. So, it encouraged me to continue holding on.”*

*(P5)*

### 3.3. Coping Strategies

Participants reported using various approaches to cope with the news of their diagnosis or how to manage their HCV-related symptoms. These approaches included avoidance/denial, a change in lifestyle, self-management, searching for information, seeking social or professional support, and seeking HCV treatment. Several strategies were used before seeking help from healthcare providers.

#### 3.3.1. Avoidance/Denial: Attempts to Ignore the Existence of Their Infection

Most participants initially ignored or dismissed their symptoms, often masking them with substance use to avoid discomfort. Over time, they learned to live with and adapt to their symptoms.

*“Well, I didn’t do anything, I was inside, so… I continued to use! (laughter) It’s not complicated! I used, and when I was high, I didn’t feel them.”*

*(P10)*

*“* *I told myself that with the little symptoms I had that it wasn’t that bad, it could stay there, you know?”*

*(P2)*

Some explained that they had difficulty accepting the diagnosis, while others were apathetic about the diagnosis or chose not to pursue a treatment course even after they received the diagnosis.

*“Since I don’t have any symptoms, it’s like I don’t give a damn, but it’s important to treat it because it can do a lot of damage. That, I know. It can lead to liver fibrosis. You can have jaundice. You can get very sick. That, I am aware of… the seriousness of the disease, but it’s like… but this time, I’m not doing well, I don’t really give a damn.”*

*(P18)*

*“I got the result, and… for a long time I kept it to myself, and I didn’t get treated.”*

*(P9)*

#### 3.3.2. Change in Lifestyle: Attempts to Prevent HCV Transmission and Avoid Judgement

Several participants mentioned that they purposely chose to isolate themselves to adapt to their HCV diagnosis. This was sometimes due to their fear of transmitting the virus to others or being judged or stigmatized by others, or due to an overall lack of energy.

*“That thing, that there is blood, and someone could have been contaminated, I have a hard time with that. Because all the same, I inject myself, there is residue left over, even if I clean myself. That’s why that ever since, I still came back in a type of isolation, still. I am still isolating myself from people.”*

*(P12)*

Some participants stated that their diagnosis led to heightened awareness of their disease, with concerted efforts to prevent onward transmission.

*“Well, first of all, I bought my syringe, and I told my boyfriend “Well, if you want one, buy one yourself because me, forget it, I… there’s no question about it, you are not taking my syringe and even less now because now, I have hepatitis C.”*

*(P6)*

The new diagnosis of HCV resulted in a shift in focus towards abstinence among a few participants, whereby their well-being was prioritized for treatment success.

*“So, it was about time I think of myself foremost instead of wanting to please my friends. That’s what was important. To be healthy for the treatment.”*

*(P5)*

*“I stopped many drugs, almost all the drugs. I just have one drug to get rid of, so I am here for that, and I understand everything, you know, mentally, I have a good mental state and a moral point of view too, the evolution of the medication is reliable, and I am pretty confident. I don’t drink booze anymore, so for my liver, I haven’t drunk at all for at least 20 years, almost 20 years. So, you know, I don’t have any problem, my liver is good, so I am encouraged. I am encouraged and I try do as much prevention as possible.”*

*(P4)*

#### 3.3.3. Self-Management: Attempts to Manage Their Infection on Their Own

Other participants coped with their new diagnosis by attempting to manage it on their own. They attempted to do so by taking over-the-counter medications, consuming more water and/or vitamins, exercising, or “allowing the body to rest.”

*“Even if I had fatigue, my physical training was important to me, so I would push myself a lot to go do it anyway. I was drinking a lot of water. They told me to drink a lot of water and vitamin C, it was something that was important.”*

*(P11)*

*“I slept a bit in the afternoon. The energy I needed; I would get it from sleep. Just the fact that I was lying down with my eyes closed, I could find the energy that my system needed to finish off my day! Just that, it helped me a lot.”*

*(P5)*

Other participants managed their disease—and the pain and anxiety it provoked—by using more drugs.

*“It lead me to use more too, in those moments, I used more because I was so anxious.”*

*(P12)*

*“[By] using, I don’t feel the pains. Well, you know, when you are not high anymore… you feel them [symptoms]. That’s life.”*

*(P10)*

#### 3.3.4. Searching for Information: Attempts to Learn More About HCV Infection

Some participants explained that their new diagnosis raised many questions for them, prompting them to search for information regarding HCV. For others, the information obtained autonomously led to them seek testing for HCV and, ultimately, their diagnosis and treatment.

*“I heard, but I did not understand. So, it was really going on the internet on my own and doing a lot of research. Doing research, trying to understand.”*

*(P12)*

*“I started asking questions. And then, starting from there, in 2019, I decided to continue doing, more research on my system. And then I passed the test, and it just confirmed it, and then I did the treatments.”*

*(P5)*

#### 3.3.5. Seeking Social Support: Attempts to Seek Emotional Support from Others

Several participants sought the support of their friends and family as part of their coping strategies. They disclosed their diagnosis and symptoms, sought their advice, or desired their company during HCV care appointments, particularly around the time of diagnosis.

*“It’s my mother that asked me to do it [screening] because she was aware that I was shooting up and that I was exchanging syringes, so she told me “Don’t do that! Don’t do that, get tested.” So, I got tested.”*

*(P6)*

*“When I went to my appointment for the answer, I asked one of my girlfriends to accompany me. At the time, I didn’t want the doctor to tell me. So, my girlfriend that accompanied me found out and it was her that gave me the answer. It was easier than if it had been a stranger telling me.”*

*(P5)*

#### 3.3.6. Seeking Professional Support

Most often, participants had sought and received professional support from harm reduction centers, community organizations for homelessness and addiction services, or community-based physicians. Only a few participants had requested help from healthcare providers while they were incarcerated.

*“I often use centers like Cactus, Spectre de rue [safe injecting sites]. So, I went to see the nurse right away, I explained to him what just happened, and he told me about hepatitis C, and we were afraid, actually, that I caught that disease again, you know? And I did some tests.”*

*(P11)*

*“As soon as I found out that I was at risk with the tattoo machine, I asked the [prison] nurse to do the tests and indeed, I was positive, so… we started the process pretty quickly.”*

*(P11)*

#### 3.3.7. Seeking HCV Treatment

Although most participants had completed HCV treatment, some explained that they deferred treatment initiation due to undesirable side effects with interferon therapy, a lack of symptoms, or their unstable lives.

*“I never went to get myself treated. I only got treated when there were the new treatments, that it was no longer Interferon, they changed and it was less painful and everything, on top of it, it was at a critical state, they told me that if I didn’t do anything, 6 months later I would be dead.”*

*(P8)*

*“So, the treatments back then scared me. That’s why I did not get myself treated. I told myself that with the little symptoms I had that it wasn’t that bad, it could stay there, you know? Also, at that time I was living day by day, I didn’t think I would go that far, you know? I was a junkie, so I thought that I could die anytime, so… it wasn’t really important for me to be cured from it.”*

*(P2)*

A few were unable to complete treatment after their initial attempts due to medication-related side effects or concomitant substance use.

*“But it’s not always easy. I stopped twice because it was making me suffer. …but I persevered, the second time, I persevered, I persevered, I didn’t stop. But the first time, I found it really hard, I said “Fuck that! I can’t do this anymore.”*

*(P6)*

*“We started a treatment with a medication, … but I wasn’t really compliant, so it didn’t amount to anything… when you’re using, you don’t necessarily take the medication. They said that I had to take them for a certain number of weeks [Hu-hum], I had them… I didn’t do it.”*

*(P12)*

Finally, some mentioned that they were still waiting for treatment to be offered to them.

*“Well, it’s because my partner was in detention, we were waiting for him to be released from prison to start the program at the same time, … we had already done the tests from the start, but they told me “We will wait for him to be released from prison, … you both are in the evolution of your disease, and we will start your program”. And each time, she gives us a different story…You know, us, we are not there to be messed around with!”*

*(P19)*

### 3.4. Situational Stimuli

Most participants relied on healthcare providers (e.g., doctors and nurses) for information about HCV. However, harm reduction centers and the internet also played an important role in the provision of information. A few participants discussed accessing information in prisons through pamphlets, group sessions, or talking to correctional officers and healthcare providers.

*“At the prison. So, she explained that to me, she gave me pamphlets on hepatitis. It’s not the nurses that gave me that, it’s the doctor that gave me that, because I told them “The nurses don’t do anything”. So, she gave me a bunch of pamphlets and then I started to protect myself better too.”*

*(P6)*

*“I find that it’s them [nurses] that do the most work. In the beginning, it was them that worked hard to show us “Hey, take care of yourself guys”, and… it was much more prevention, but they gave a lot of information also for what lied ahead, and all that and the programs that followed, and they also gave a lot of hope to be cured from this disease. But I would say, very much, the nurses did a lot in that,”*

*(P11)*

*“I asked the guards, and… I asked for all the information that I could get, and they brought it to me. I asked to go on the Internet at the library. And they brought me… I was lucky.”*

*(P14)*

Other sources of information included friends, family, and street workers.

*“How I obtain the information? By … the street workers, the people that I meet that have hepatitis, but it’s not always good information. On the streets, they can say anything, and I will always get my information from people.”*

*(P17)*

*“It’s my drug dealer that explained what the disease was and that there was a treatment for it.”*

*(P3)*

## 4. Discussion

This study explored the perceptions of HCV and its management among PWEI in Quebec provincial prisons who had been released into the community in the preceding 12 months, using an established social cognition model, the CS-SRM. Participants primarily reported delays in seeking initial HCV care, including screening, due to non-specific symptoms and stigma related to injection drug use and/or associated with incarceration. Following diagnosis, most participants reported experiencing fear and attempted several coping strategies during the course of illness (e.g., self-management, seeking social support, and seeking information) before visiting a healthcare provider. While knowledge of HCV among PWEI appears to be improving over time, holistic education that involves correctional officers and healthcare providers may help overcome stigma, address fears, and improve awareness of both patient- and occupational-level benefits of HCV testing and treatment of PWEI.

Delays in seeking HCV care were primarily due to non-specific symptoms and associated coping mechanisms following diagnosis. Generalized fatigue and gastrointestinal symptoms not specific to chronic HCV infection, or no symptoms at all, made it difficult to consider a diagnosis of HCV and/or seek care. Furthermore, some participants initially attributed their symptoms to food poisoning, other infections, or medication side effects, as has been found in other studies [49,50,51]. Following the diagnosis of HCV, many participants isolated themselves from friends and the public. Some coped by using drugs to mask symptoms, further compromising their ability to acknowledge their infection. Most often, however, self-isolation was a strategy to avoid experiencing prejudice and stigma by others and/or to reduce the fear of transmitting the virus to others [30,52].

Participants reported experiencing several forms of stigma, including but not limited to stigma related to having HCV, injection drug use, and/or associated with incarceration. Stigma contributed to diminished feelings of self-worth as a result of being viewed as a “junkie”, a “dishonest person”, or even “a criminal”, findings that are corroborated elsewhere [52]. Internalized stigma—that is, when social attitudes about people who inject drugs and are temporarily housed in prison become part of a person’s understanding of themselves and their sense of self-worth—may also impact people in prison [52] and subsequent health-seeking behaviors. Internalized stigma has been associated with poor health outcomes and healthcare-system engagement [53], pointing to the urgent need to offer education and training to facilitate destigmatized approaches to substance use and HCV that still promote institutional safety and security during incarceration.

Participants reported several fears following their HCV diagnosis. They feared transmitting the virus to others, worried about their life expectancy and the well-being of their children, and, consequently, limited their inter-personal relationships [54]. In fact, studies have demonstrated that PWEI are motivated to start HCV treatment as an act of reciprocity to their family and to improve inter-personal relationships [55,56,57]. This not only highlights the need to raise awareness about HCV prevention and transmission among PWEI but may also assist healthcare providers in better understanding how to deliver information about HCV, recognizing that PWEIs’ fears can be allayed with early treatment initiation. Simplifying and streamlining the pre-treatment clinical evaluation and workup within a single visit in carceral settings has been shown to be associated with high treatment uptake in prison and high levels of acceptability among PWEI [34,58]. While immediate treatment initiation is complicated in prison due to short incarceration durations and challenges with movement, it is important to recognize that this approach may have an important positive psychological impact on PWEI, an area that requires further research.

Encouragingly, participants reported an improvement in HCV knowledge over time—albeit still plagued with misconceptions—which resulted in changes in their perception of HCV and its management. Information about HCV was provided to PWEI through various mechanisms. Many had received verbal information from their healthcare providers during screening and/or treatment; however, some struggled with understanding and processing due to their initial shock and disbelief. Some participants accessed information on HCV in prison through pamphlets and peer-led group sessions, as well as through correctional officers. This suggests that access to HCV information for PWEI may be improving. However, gaps in HCV health literacy (knowledge, attitudes, and competencies) among key sectoral sub-populations (healthcare providers, correctional officers, and PWEI) have been identified as major barriers to enhanced HCV care for PWEI [59,60]. Consequently, whole-of-sector education that includes correctional officers and healthcare providers may help overcome stigma and improve awareness of both the patient- and occupational-level benefits of HCV testing and treatment of PWEI [60,61,62]. Due to highly entrenched prison subcultures, peers may be considered part of an attractive and cost-effective educational strategy for PWEI [63] which may lead to meaningful changes in the way PWEI engage in HCV-related care [64]. Finally, many participants relied on community organizations, such as safe injection sites, as their primary sources of information. As these are often frequented by PWEI following release, enhancing communities’ organizational capacity to provide education and/or HCV screening (including other sexually transmitted and blood-borne infections) will be key to HCV elimination among this population [65].

Finally, prior to this study, little was known about how people with chronic HCV cope following their diagnosis [66], and nothing was known about how PWEI do so. Participants reported various coping strategies following their HCV diagnosis and to describe how they managed their HCV-related symptoms. Most of these coping strategies consisted of seeking information to improve health literacy; instrumental and emotional support from family, friends and professionals; and HCV treatment to accelerate cure. Conversely, and despite the associated risks, some resorted to avoidance or denial as a coping mechanism whereby increased drug use helped numb symptoms and the pain and anxiety provoked by the new diagnosis. This coping strategy was also found in a recent study with people who inject drugs in Montréal [46]. Understanding coping strategies among PWEI following a new HCV diagnosis is important, as it can assist healthcare providers in better understanding how best to deliver information and determine how closely the patient needs to be seen in follow-up depending on hypothesized coping strategies.

Our study has limitations. First, our study was conducted during the height of the COVID-19 pandemic in Canada, when all prison research was halted to reduce the risk of SARS-CoV-2 outbreaks in prison. While we would have preferred to interview people in prison, we instead chose to interview people with recent lived experience of incarceration. This means that all of our results may not be generalizable to the incarcerated population in Quebec, as participants were likely reflecting on experiences during incarceration and post-release. Furthermore, given overlapping risk factors for HCV acquisition, it is not surprising that similar findings were observed in a recent study with people who inject drugs in Montréal [46]. Second, as all interviews were conducted in French, they required translation to English for analysis. In doing so, certain meanings and concepts may have been lost in translation. That being said, both interviewers were fluent in both English and French. Third, as we collected minimal sociodemographic characteristics of participants, our findings may not be representative of the broader provincially incarcerated population in Quebec or Canada. Fourth, while the CS-SRM separates cognitive and emotional representations, our findings demonstrate that these domains often overlap. For example, beliefs about HCV consequences, like social isolation or fear of transmission, blend cognitive evaluations with emotional responses, emphasizing the need for a nuanced understanding of illness perceptions. Fifth, the small sample size and heterogeneity in participants’ HCV treatment status restrict our ability to draw conclusions about differences in experiences based on treatment outcomes, such as patterns of social, sexual, or emotional isolation. Additional research with larger and more stratified samples is needed to investigate these patterns more systematically. Finally, as with all qualitative research, social desirability bias may have been introduced. Conversely, to our knowledge, this is the first study to characterize perceptions of HCV and its management, specifically with PWEI, using the CS-SRM.

## 5. Conclusions

Despite advances in HCV treatment, PWEI continue to experience many forms of stigma and fear surrounding their HCV diagnosis, resulting in delayed HCV care. These findings highlight the urgent need for whole-of-sector HCV education (correctional officers, healthcare workers, and PWEI), and they simultaneously provide insights into how prison- or community-based healthcare providers can better understand and utilize HCV illness perceptions to evaluate willingness to engage in HCV care and treatment among PWEI.

## Figures and Tables

**Table 1:** Common Sense Self-Regulation Model (CS-SRM) dimensions and definitions, and example questions from the interview guide.

**1. Cognitive illness representations**	1.1 Identity	The way symptoms are perceived and ascribed to the illness, including the assigned label for the illness itself.	What were your symptoms? If you have not had any symptoms, do you know what the possible symptoms are?
	1.2. Timeline	Beliefs concerning the speed of onset, duration, and variations in the illness, such as whether it is acute or chronic.	How long has it been since you first experienced the symptoms/diagnosed? How long did your symptoms last? Do/did they fluctuate?
	1.3. Cause	Beliefs regarding the factors leading to the illness, such as genetics, infection, diet, aging, or other contributing elements.	How do you think you acquired HCV, or what causes it?
	1.4. Consequences	Beliefs about the anticipated impact of the illness on various aspects of life, including work, family, and personal relationships.	What are/were the consequences of HCV infection for you?
	1.5. Controllability/curability	Beliefs concerning the controllability/curability of the illness.	Do you think your HCV infection can be cured? How? If not, can HCV infection be controlled? Is there anything that can improve your symptoms?
	1.6. Coherence	The patient’s comprehension of the illness and how they construct meaning around it.	How well do you feel you understand your illness and treatment options? Does it all make sense to you?
**2. Emotional illness representations**		Beliefs/perceptions related to the emotional effects of the illness.	How does/did this illness and its symptoms make/made you feel? Does it make you angry, scared, upset, or depressed? What are your most important concerns about it?
**3. Coping strategies**		Approaches used to deal with the illness, adjusted based on success or failure in managing previous episodes of illness.	How do/did you usually manage your symptoms, if any? Does/did it help?
**4. Situational stimuli/sources of information**		Lay information stored in memory, insights from expert sources, and experienced somatic and symptomatic information.	How do/did you get information about HCV and its treatment/care? Where do you usually look for information?

## Data Availability

Study data and materials may be shared following a request to the corresponding author.
